# The Role of Vesicle Trafficking Defects in the Pathogenesis of Prion and Prion-Like Disorders

**DOI:** 10.3390/ijms21197016

**Published:** 2020-09-23

**Authors:** Pearl Cherry, Sabine Gilch

**Affiliations:** 1 Calgary Prion Research Unit, Department of Comparative Biology and Experimental Medicine, Faculty of Veterinary Medicine, University of Calgary, Calgary, AB T2N 4Z6, Canada; pearl.cherry@ucalgary.ca; 2 Hotchkiss Brain Institute, University of Calgary, Calgary, AB T2N 4Z6, Canada

**Keywords:** prion, prion diseases, prion-like diseases, vesicular trafficking, Rab7, therapy

## Abstract

Prion diseases are fatal and transmissible neurodegenerative diseases in which the cellular form of the prion protein ‘PrP^c^’, misfolds into an infectious and aggregation prone isoform termed PrP^Sc^, which is the primary component of prions. Many neurodegenerative diseases, like Alzheimer’s disease, Parkinson’s disease, and polyglutamine diseases, such as Huntington’s disease, are considered prion-like disorders because of the common characteristics in the propagation and spreading of misfolded proteins that they share with the prion diseases. Unlike prion diseases, these are non-infectious outside experimental settings. Many vesicular trafficking impairments, which are observed in prion and prion-like disorders, favor the accumulation of the pathogenic amyloid aggregates. In addition, many of the vesicular trafficking impairments that arise in these diseases, turn out to be further aggravating factors. This review offers an insight into the currently known vesicular trafficking defects in these neurodegenerative diseases and their implications on disease progression. These findings suggest that these impaired trafficking pathways may represent similar therapeutic targets in these classes of neurodegenerative disorders.

## 1. Prion Diseases

Prion diseases or transmissible spongiform encephalopathies are fatal spongiform neurodegenerative disorders in humans and in other mammals. The classic hallmark of prion diseases is the deposition of amyloid plaques in the brain, which leads to progressive neuronal loss, spongiosis, and astrogliosis [1,2]. They are caused by prions, proteinaceous infectious particles arising upon misfolding of the ubiquitously expressed cellular protein named PrP^c^ into an insoluble and aggregation-prone conformer named PrP^Sc^ [3,4]. Prion diseases in humans can have three different etiologies—sporadic, familial or acquired by infection [5,6]. Most cases of human prion diseases are comprised of sporadic Creutzfeldt-Jakob disease (sCJD). Gerstmann Sträussler Scheinker syndrome and fatal familial insomnia (FFI) are inheritable forms of the disease, linked to specific mutations in the *PRNP* gene encoding the prion protein, e.g., P102L and D178N in combination with methionine at codon 129, respectively. Kuru, identified in some tribes in Papua New Guinea that practiced cannibalism, variant CJD (vCJD), and iatrogenic CJD are forms of the disease acquired by infection. Iatrogenic CJD transmissions have been described upon treatment with cadaveric growth hormone preparations contaminated with prions, as well as by corneal transplants from prion infected individuals [7,8]. Horizontal transmission of vCJD in humans has also been reported through blood transfusions [9]. The major clinical symptoms associated with prion diseases in humans include rapid cognitive decline and ataxia [10].

Historically, the first ever reported prion disease, as early as in the 18th century, is scrapie in sheep and goats [11]. Other animal prion diseases include bovine spongiform encephalopathy (BSE) in cattle and chronic wasting diseases in cervids, transmissible mink encephalopathy, feline spongiform encephalopathy, and the recently described camel prion disease [12,13]. Clinical signs vary depending on the prion strain and the genetic background of the animal but generally involve ataxia, hyperexcitability, and tremors. The zoonotic potential of prion diseases was manifested through the transmission of BSE to humans, giving rise to a novel form of human prion disease called vCJD, in the 1990s, mostly in the UK, claiming more than 200 lives [14,15].

## 2. Prion Conversion

The characteristic difference between PrP^c^ and PrP^Sc^ is that PrP^c^ is predominantly composed of alpha helices, while the infectious isoform PrP^Sc^ is composed of β-sheets [16]. The high β-sheet content accounts for the propensity of PrP^Sc^ to aggregate and its partial resistance to proteinase K digestion and cellular proteases. It has been proposed that the infectious PrP^Sc^ can acquire various conformational arrangements thought to be associated with the prion strain phenomenon [17]. These strains can transmit their particular conformation to the host’s PrP^c^ leading to distinct disease features [18].

The mechanism of PrP^c^ to PrP^Sc^ conversion is still ambiguous. Among the proposed models to explain the conversion of PrP^c^ to PrP^Sc^, the most favored model is the nucleated polymerization model [19]. The pathogenic conformer PrP^Sc^ forms oligomers, which act as the seed to convert the endogenous PrP^c^ to its infectious conformer which is incorporated into the growing protofibrils and eventually into amyloid-like fibrils. As the fibrils grow, they break into smaller seeds which catalyze additional conversion and elongation of PrP^Sc^ fibrils. Hence, fibril fragmentation is the rate limiting step in the pathological process of prion replication [20,21]. Spagnolli et. al. put forward a template-assisted conversion model, where PrP^Sc^ exists in a 4-rung β-solenoid structure which acts as the template inducing the self-catalytic conversion of PrP^c^ monomers into PrP^Sc^ protofibrils [22]. Many arguments exist in the field favoring the possibility of a cofactor that could aid in prion replication but, so far, no such absolute player has been identified [23,24,25]. The conundrum still remains as the synthesis of infectious prions in the absence of any co-factor has, so far, not been attained experimentally. However, endogenous phospholipids, such as phosphatidylethanolamine, and polyanions, like RNA molecules, have been used for in vitro generation of high titer infectious prions [26,27].

Although clinically and etiologically very diverse, a common characteristic between many neurodegenerative diseases is their causal association with protein misfolding and the aggregation of the respective proteins, such as α-synuclein in Parkinson’s disease (PD), β-amyloid, and tau in Alzheimer’s disease (AD), and huntingtin in Huntington’s diseases (HD), which leads to an impaired protein homeostasis [28]. Unlike these common neurodegenerative disorders, prion diseases occupy a unique position as they can be acquired by natural infection. Despite that the pathological proteins implicated in these diseases are very different, they all exhibit similar molecular mechanisms of pathological conversion, whereby the misfolded form of the protein can act as a seed and catalyze conformational changes of the endogenous protein, fibril elongation and formation of amyloid plaques [29,30,31]. Therefore, these diseases are referred to as prion-like disorders [32]. They also share other commonalities with prion diseases, in particular, cell-to-cell spread of misfolded protein aggregates. This transmission can take place by passive release of the protein aggregates via cell death or by vesicle-mediated exocytosis or exosomes [33,34]. Evidence also shows the existence of direct propagation from cell to cell by tunneling nanotubes [35,36].

## 3. PrP^c^ Trafficking and Its Role in PrP^Sc^ Conversion

The cellular prion protein PrP^c^ is a glycosyl-phosphatidyl-inositol-(GPI) anchored glycoprotein [37]. It is ubiquitously expressed and structurally highly conserved in vertebrates [38]. Endogenous expression of PrP^c^ is highest in neurons, primarily at the synapses, and is more abundant in lymphoid tissue as compared to other tissues. Sub-cellularly, PrP^c^ is predominantly enriched at the plasma membrane in the lipid rafts [39]. It consists of about 250 amino acids depending on the species, with an *N*-terminal signal peptide that targets it to the lumen of the endoplasmic reticulum (ER) in a co-translational manner. The *C*-terminal of the protein is modified by the addition of the GPI-anchor, mediated by a *C*-terminal signal peptide which is cleaved off. The mature form of PrP^c^ that is released from the ER consists of about 209 amino acids, harbors one disulfide bond and is *N*-glycosylated at two sites. PrP^c^ has a highly conserved hydrophobic domain that connects the flexible unstructured *N*-terminal domain containing five to six proline and glycine rich copper binding octapeptide repeats with the globular *C*-terminal domain containing three alpha helices and two short beta strands [40,41]. Like other secretory proteins, PrP^c^ is transported through the Golgi complex, undergoing extensive post translational modifications, such as sialylation, and is anchored to the outer leaflet of the plasma membrane [42,43]. PrP^c^ internalization can occur via clathrin- or caveolae-mediated endocytosis. Clathrin-mediated endocytosis is mediated by non-canonical interaction with the clathrin adaptable proteins, such as LRP1 (low density lipoprotein receptor related protein 1), in which the *N*-terminal of PrP^c^ is crucial for internalization [44,45,46]. It can then be recycled back to the plasma membrane via recycling endosomes [44]. Caveolae-mediated endocytosis of PrP^c^ has been observed in microglia and neuroblastoma cell lines and is stimulated by copper binding [47]. PrP^c^ ends up in lysosomes, where it is degraded [48].

A seminal study showed that *Prnp* knockout mice are resistant to scrapie infection, which indicates that the expression of PrP^c^ is the crucial factor necessary for prion propagation [49,50,51]. Furthermore, it is known that the two isoforms directly interact to result in the re-folding of PrP^c^ to PrP^Sc^. Therefore, subcellular trafficking and co-localization of both isoforms in specific subcellular compartments are important to facilitate their physical contact. PrP^Sc^ is localized at the plasma membrane and in different intracellular compartments including early endosomes, recycling endosomes, multi vesicular bodies (MVBs)/late endosomes, lysosomes, and perinuclear Golgi regions, coherent with the localization of PrP^c^ [52,53]. Altering the trafficking of PrP^c^ and PrP^Sc^, hence preventing its interactions at the sites of conversions, can be deployed to reduce prion propagation.

**Figure 1:**
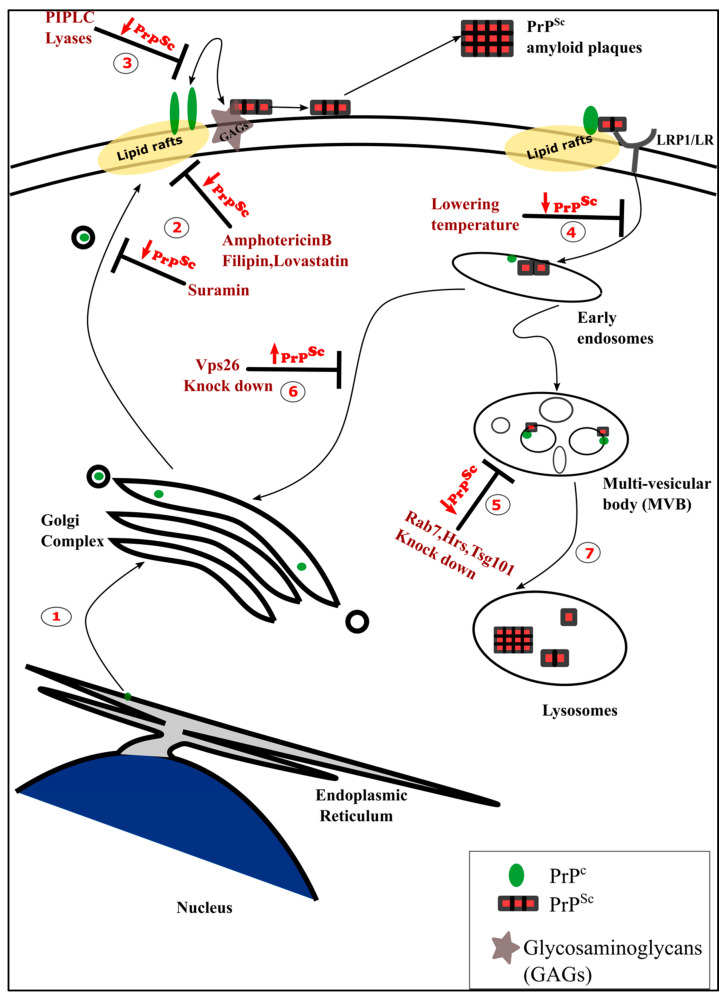
Trafficking of a cellular form of the prion protein (PrP^c^) and an infectious and aggregation prone isoform (PrP^Sc^) and the potential inhibitors of the pathogenic conversion at the different stages of trafficking. (**1**) Cellular PrP^c^ is synthesized in the endoplamic reticulum (ER), transported to the Golgi complex, and then is finally localized at the plasma membrane in the lipid rafts. (**2**) Preventing the surface localization or the accessibility of PrP^c^ at the plasma membrane (PM) is one strategy adopted to reduce PrP^Sc^ levels. This can be done by treating cells with suramin, which retains the PrP^c^ in the Golgi or by abrogating the proper lipid raft formation by treating with amphotericin B, filipin, or lovastatin, which prevent the localization of PrP^c^ in the lipid rafts and subsequently prevent PrP^Sc^ formation. (**3**). Phosphoinositide phospholipase C (PIPLC)-mediated cleavage of PrP^c^ or the removal of glycosaminoglycans (GAGs) by lyases, reduce PrP^Sc^ levels. (**4**). Preventing the endocytosis of PrP^c^/PrP^Sc^ by lowering the temperature and (**5**) preventing the maturation of the multi-vesicular bodies can also reduce PrP^Sc^ propagation. (**6**) In contrast, inhibiting the retrograde trafficking to Golgi increases PrP^Sc^ levels. (**7**) Eventually, PrP^Sc^ accumulates in lysosomes where it is partially degraded.

Previous studies using mostly persistently prion-infected neuronal cell lines have already indicated that the cell surface localization of PrP^c^, cholesterol levels, and the endocytic pathway are important for prion conversion (Figure 1). Lowering the temperature to inhibit the endocytosis of PrP^c^ reduces PrP^Sc^ levels, manifesting the role of the endocytic pathway in prion conversion [54]. Preventing the lipid raft localization of PrP^c^ by modifying the *C*-terminal targeting sequence, lowering cellular cholesterol levels, or retaining it in the post ER-Golgi compartment reduces PrP^Sc^ propagation, implicating the necessity of the localization of PrP^c^ in the lipid rafts for PrP^Sc^ propagation. Cleaving off the surface exposed PrP^c^ with the enzyme phosphatidylinositol-specific phospholipase C (PIPLC) also inhibits prion conversion by interfering with PrP^c^–PrP^Sc^ interactions at the plasma membrane [55,56,57]. Interestingly, transgenic mice expressing a secreted form of PrP^c^ lacking the GPI-anchor accumulate infectious prion plaques in the brain without developing the characteristic clinical scrapie symptoms [58]. Only when higher levels of the GPI-anchorless PrP are expressed, these mice develop prion disease with unique clinical signs [59]. The co-expression of GPI-anchored and GPI-anchorless PrP^c^ accelerates clinical disease, indicating that the GPI-mediated membrane attachment of PrP^c^ may be required for transmitting the toxic signals [58]. PrP^c^ and PrP^Sc^ interacting receptors, like glycosaminoglycans (GAGs), laminin receptor precursor (LRP/LR), and LRP1, have a prominent role in the conversion process. It has been demonstrated that cultured cells harboring reduced amounts or modified GAGs, such as heparin sulfate, are resistant to primary prion infection [60,61]. Interfering with lipid raft formation by drugs, such as lovastatin, filipin, and amphotericin B, was capable of reducing PrP^Sc^ levels in neuroblastoma cell lines persistently infected with scrapie prion strains, thus emphasizing the role of lipid rafts in PrP^Sc^ conversion [47,55,62]. Altering the sub-cellular localization of cholesterol from the plasma membrane to the lysosomes by treating prion-infected neuroblastoma cells with the drug U18666A reduces PrP^Sc^ levels. This reduction is restored by rescuing the trafficking of cholesterol to the trans-Golgi network and subsequently to the plasma membrane by over-expression of the Rab9 protein [63].

Several studies modulating endocytic vesicular trafficking demonstrate a reduction in PrP^Sc^ levels suggesting multivesicular bodies (MVBs) and endocytic-recycling compartments (ERC) as the intracellular sites of pathogenic prion conversion [53,64,65,66]. Yim et al. [65] show that the knock-down of proteins required for the maturation of MVBs, such as Rab7, and components of the endosomal sorting complexes required for transport (ESCRT) machinery, such as Hrs and Tsg101, resulted in a reduction of PrP^Sc^ levels. Knock-down of proteins that are part of the retromer complex, such as vacuolar protein sorting-associated protein 26 (Vps 26), which retrieves cargo away from MVBs resulted in increased PrP^Sc^ levels, supporting the conclusion that MVBs could be the site of PrP^Sc^ conversion [65]. Manipulating the levels of proteins that are involved in the endocytic recycling also alters PrP^Sc^ levels, suggesting that the pathogenic conversion of PrP^c^ can also occur here. Over-expression of dominant negative forms of Rab4 involved in rapid recycling of early endosomes to the plasma membrane does not affect PrP^Sc^, suggesting that this pathway is dispensable for conversion. However, overexpression of a dominant-negative Rab11, a protein critical for slow cargo recycling to the plasma membrane via recycling endosome increases PrP^Sc^ levels indicating that the endocytic recycling compartment (ERC) is a site of prion conversion [53,67]. The mode of PrP^Sc^ internalization differs depending on the prion strain and the stage of infection [68], raising the question whether all prion strains adopt to the same site for prion conversion. Eventually, the majority of PrP^Sc^ accumulates in the lysosomes, where it is partially degraded [53,64].

There is still a knowledge gap about how and what factors could be involved in the cellular conversion of PrP^c^ into the infectious PrP^Sc^ isoform. The evidence of the involvement of more than one sub-cellular site in prion conversion as discussed above also questions whether this conversion is a sequential process, where the byproducts of the processing of PrP^c^/PrP^Sc^ at the plasma membrane, MVBs and ERC act hand in hand in the formation of the non-degradable PrP^Sc^ that accumulates in the lysosomes. Furthermore, the existence of different strains makes it challenging to define a specific site of conversion, considering the likely existence of strain-specific preferences. In vivo, there is not always a strong correlation between PrP^Sc^ deposition and cell death. Follicular dendritic cells in the lymphoid tissues [69] and astrocytes [70] can efficiently amplify PrP^Sc^ but does not lead to their cell death. In fact, depleting, specifically, the neuronal prion protein in transgenic mice can prevent PrP^Sc^ deposition in the neurons and hinder the clinical manifestations of the disease, such as neurodegeneration, and spongiform changes, despite an effective propagation of PrP^Sc^ in other non-neuronal cell lines [71].

Most of the discussed studies were performed in persistently or acutely prion-infected cell lines of neuronal, but also of non-neuronal, origin, which have been established as valuable models to study the cell biology of prion proteins, while, only in a few cases, these findings were confirmed using primary neurons. Limitations of the cell line models of prion propagation include that they do not exhibit neurodegeneration and do not reflect the diversity of neurons as observed in the brain.

Tissue tropism of prion strains is another area that is difficult to study with the existing cell models. This phenomenon is exemplified by the drowsy and the hyper strains of hamster-adapted transmissible mink encephalopathy [72]. The drowsy strain is more restricted to the central nervous system (CNS) and has a lower capability to propagate in the lymphoreticular cells, probably because of the existence of a more efficient clearance mechanism for this strain in the lymphoreticular system [73,74]. However, the hyper strain has a more diverse distribution and can be detected in the CNS, lymphoreticular system, skeletal muscle, nasal secretions, and blood [75,76,77].

Currently, no lymphatic cell-type model exists to study prion replication. Such a model representing another major tissue type propagating prions in an infected host would be desirable to investigate and compare the requirements for and the consequences of prion propagation in different cell types.

## 4. Consequence of Prion Infection on Vesicular Trafficking

While the above studies demonstrate that the transport and the localization of PrP^c^ and PrP^Sc^ to specific sub-cellular compartments are critical for prion propagation, there is evidence that prion infection or the presence of PrP^Sc^ aggregates in intracellular vesicles negatively impact and alter endocytic vesicle trafficking. 

### 4.1. Rab GTPases—Coordinators of Vesicular Trafficking

Endocytic vesicular trafficking includes all processes from the internalization of cargo in the form of vesicles from the plasma membrane to the sorting and the transport of these vesicles into different endocytic compartments—to be retrieved by the Golgi complex, or recycled back to the plasma membrane or transported to the lysosomes for degradation. The master regulators in these pathways which help in the trafficking of vesicular cargo to different endocytic compartments and give specific organellar identity to these endocytic compartments are a family of proteins called the Rab GTPases. Rab GTPases, in coordination with their effector proteins and the Sar/Arf family of GTPases, regulate cargo selection and vesicle formation [78].

**Figure 2:**
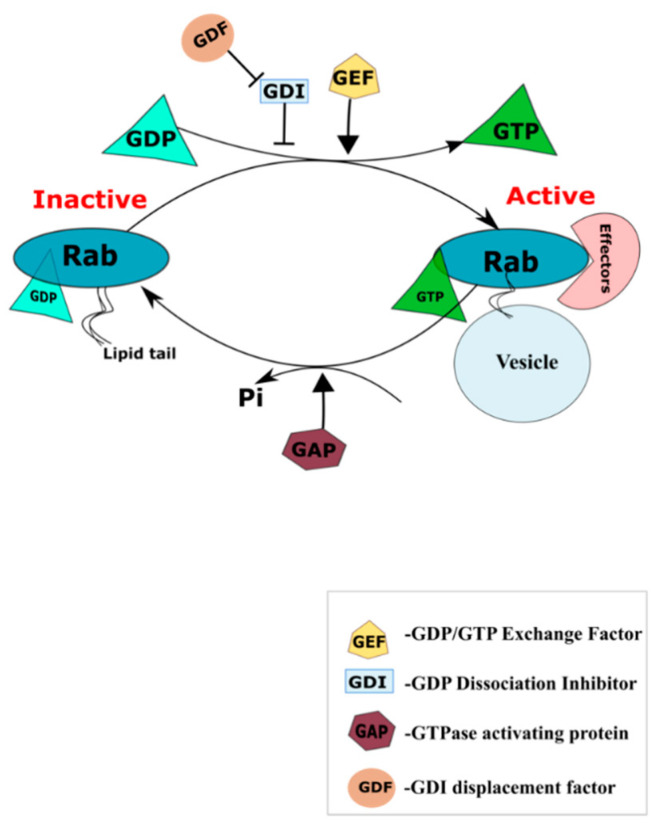
Rab activation cycle. GDP/GTP exchange factors (GEFs) mediate Rab activation by replacing the GDP with GTP. The GTP bound Rabs can interact with their effector proteins and mediate vesicular trafficking. Rabs are in an inactive state when bound to GDP, and this involves the hydrolysis of GTP by GTPase activating protein (GAP). The GDP bound Rab7 is prevented from further activation cycle by their association with GDP dissociation inhibitor (GDI). GDI displacement factor (GDF) displaces GDI and makes it available for another cycle of activation.

Rabs are monomeric GTPases and the largest branch of proteins in the Ras superfamily of GTPases. They have a G-domain which can bind guanosine nucleotides and the *C*-terminal membrane targeting domain is prenylated. Prenylation enables reversible insertion of these proteins into membranes, allowing them to exert their function. Most organelles in the endo-membrane system, like the ER, Golgi, endosomes, and lysosomes, are defined by the presence of characteristic Rab proteins on their surfaces facing the cytosol which facilitate their interaction with other organelle-specific proteins. These include but are not limited to Rab-bound motor proteins and Rab-bound tethering molecules [79,80]. Rab proteins exert their function when activated by binding to GTP, which is mediated by the guanine nucleotide exchange factors (GEFs). The GEF-mediated nucleotide exchange is the primary event that triggers the localization of Rabs to the membranes facilitating interaction with other effector proteins. They are inactive when in a GDP-bound state, and inactivation involves the hydrolysis of GTP through their inherent GTPase activity, which is enhanced by the guanine activating factors (GAP). The GDP-bound Rab proteins are extracted from the membrane and prevented from further activation cycles by their association with GDP Dissociation Inhibitor (GDI) until the GDI displacement factor (GDF) displaces the GDIs and recruits the Rab protein to the membrane for another cycle of activation [81] (Figure 2).

### 4.2. Rab7

**Figure 3:**
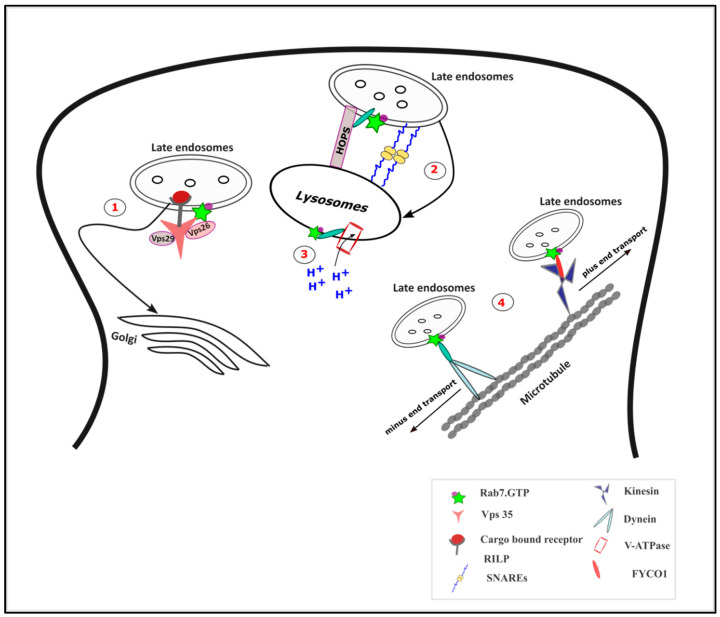
Functions of Rab7 in vesicular trafficking: (**1**) The retrograde transport of cargo to the Golgi complex is mediated by Rab7 via its interaction with the subunits of retromer complex especially vacuolar protein sorting-associated protein 35 (Vps 35). (**2**) Rab7-interacting lysosomal protein (RILP) interaction with the Homotypic fusion and Protein Sorting (HOPS) complex mediates the endosome-lysosome fusion along with the SNAREs. (**3**) The Rab7-RILP complex regulates the function of Vacuolar-ATPase (V-ATPase) and hence in lysosomal acidification. (**4**) Rab7, by interacting with RILP, mediates the minus end transport of the late endosomes, while its interaction with FYVE and coiled-coil domain autophagy adaptor-1 (FYCO1) mediates the plus end directed transport.

In the endo-lysosomal system, early endosomes are marked by the presence of Rab5 and as they mature to late endosomes and lysosomes, characterized by the increased luminal acidification; there is also an increased recruitment of Rab7 which displaces Rab5 [82]. Rab7 is the main player in the late endosomal maturation and transport along with retromer regulation (Figure 3). The Rab7 effector Rab7 interacting lysosomal protein (RILP) interacts with the HOPS (Homotypic fusion and Protein Sorting) complex to mediate endosome-lysosome fusion. The Rab7-RILP interaction also regulates the assembly and the function of the Vacuolar-ATPase (V-ATPase) for lysosomal acidification. Rab7, by differentially interacting with RILP and FYVE and coiled-coil domain autophagy adaptor-1 (FYCO1), mediates the minus and plus end directed transport of late endosomes, respectively. The retrograde transport of transmembrane cargo to the trans-Golgi network is also regulated by the interaction between Rab7 and sub-units of the retromer complex especially Vps 35 [83].

It is reasonable to connote that the presence of PrP^Sc^ aggregates in the endocytic vesicles could disturb the vesicular trafficking pathways in the affected cells. Even though the importance of subcellular trafficking of PrP^c^ and PrP^Sc^ in sustaining prion infection has been demonstrated by numerous studies, the impact of prion infection on vesicle trafficking is less clear. Here, we showcase some of the known trafficking impairments identified in prion diseases and draw a contrast on similar trafficking defects known in other prion-like disorders. More importantly, it is a tempting question whether the trafficking defects observed are the cause or the effect of PrP^Sc^ propagation and neuronal cell death in these neurodegenerative diseases.

## 5. Prion Related Vesicular Trafficking Defects

**Figure 4:**
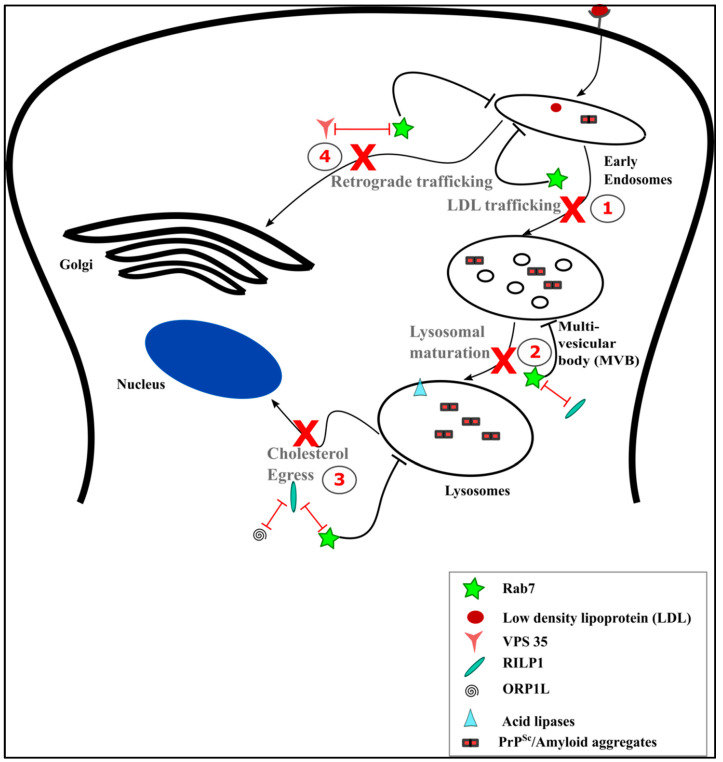
Potential Rab7 mediated impairments upon prion infection. Active levels of Rab7 are reduced upon prion infection which implicates an impaired function of Rab7 in the endocytic vesicular trafficking. The following functions of Rab7 in trafficking could be impaired in prion infection: (**1**) LDL trafficking and consequently cholesterol metabolism; (**2**) the interaction of Rab7 with RILP1, resulting in decreased lysosomal maturation; (**3**) Rab7-RILP interaction with oxysterol-binding protein-related protein 1L ORP1L, resulting in inefficient cholesterol egress from the lysosomes; and (**4**) Rab7 interaction with Vps 35 resulting in defective retrograde trafficking.

Vesicular trafficking defects associated with pathological changes have been very clearly observed in CJD brains in the histopathological studies—such as enlargement of Rab5 positive early endosomes in regions with mild pathology and enlarged cathepsin D and B immunopositive lysosomes in regions with moderate pathology [84]. Enlarged early endosomes may be due to the accumulation of vesicular cargoes because of impaired exit pathways towards retrograde trafficking and lysosomal degradation. Shim et al. show that the amount of membrane associated Rab7 is significantly reduced in prion infected neuronal cell lines [85]. This reduction in Rab7 could in turn have implications on its physiological functions in the cell where it acts as a key regulator in early-to-late endosomal transition, cargo sorting, lysosomal maturation, neurotrophin transport, lipid metabolism, and autophagy, to name a few of its direct functions [83]. The impaired lysosomal acidification and reduction in the efficiency of lysosomal degradation as reflected by the increased half-life of epidermal growth factor receptor (EGFR) upon prion infection can be linked to the reduced membrane associated Rab7 [85]. These findings further indicate a potential loss of other Rab7 functions, as well in prion-infected neurons (Figure 4).

Another vesicular trafficking pathway affected by prion infection is the retrograde trafficking as observed from the analysis of RNA expression profiles in sporadic CJD brains implicating down regulated gene expression, as well as reduced protein levels of vacuolar protein sorting-associated protein 35 (Vps 35), a subunit of the retromer complex [86]. Prion infection also disturbs post-Golgi trafficking of proteins, like attractin and the α-subunit of insulin receptor (IRα), in 22L and RML/chandler prion infected neuroblastoma cell lines [87]. Reduced levels of functional Rab11 protein were observed in cell lines expressing a mutant prion protein associated with FFI. This was indeed due to the elevated expression levels of Rab GDI, causing an increased fraction of inactive cytosolic Rab11. The enlarged Golgi morphology observed in these mutant prion protein expressing cell lines reflect dysregulated post-Golgi trafficking, a process that is mediated by Rab11 [88].

Studies also show that Rab7 mediated axonal retrograde trafficking of cargo is impaired in motor neurons of prion infected mice. These impairments were observed at the onset of the clinical disease and were not related to neuronal death, allowing the correlation of this phenotype with the clinical signs of the disease, such as hindlimb paralysis and ataxia [89]. Rab5 and Rab7 control the endocytic sorting of neurotrophins, such as brain derived neurotrophic factor (BDNF), along the axonal retrograde pathway [90]. Since prion-infected mice do show a selective impairment in the axonal retrograde vesicle transport, which transport neurotrophins and their receptors, it is highly probable that the retrograde trafficking of BDNF in prion infection is affected and could be a factor leading to neurodegeneration [85,89].

## 6. Vesicular Trafficking Defects in Prion-Like Disorders

As in the case of prion diseases, one of the earliest characteristics associated with the pre-clinical stages of AD, even before any detectable amount of amyloid deposition is the enlargement of Rab5-positive early endosomes. The pro-protease and mature forms of cathepsin B and D were also detected in these enlarged endosomes, along with cation dependent mannose 6 phosphate receptor (MPR) depicting a failed sorting and trafficking of vesicular cargo along the endo-lysosomal pathway in these AD cases [91,92].

Another striking similarity is the Rab7 loss of function that has been implicated in the pathogenesis of prion diseases. It is interesting to note here that Rab7 recruits the retromer complex through its direct interaction with the *N*-terminal of Vps 35 [93], and another common trait observed in PD, AD, and sCJD are reduced Vps 35 levels. Mutations in leucine-rich repeat kinase 2 (LRRK2) are associated with late onset PD [94]. Pathogenic LRRK2 reduces Rab7 activity and Vps 35 levels, and consequently delays the transit of cargo from the late endosomes to the lysosomes, resulting in an impaired EGFR degradation [95,96]. Rab7 activation and consequently its membrane association are impaired in autosomal recessive early onset PD linked to a mutation in the parkin gene. This leads to a decreased endosomal tubulation and an impaired retromer pathway due to the lack of recruitment of Vps 35 and sorting nexin 1 (SNX1) in primary dermal fibroblast cells from PD patients carrying a homozygous mutation in the parkin gene [97]. Similarly, Vps 35 protein levels are reduced in post-mortem AD brains [98]. In vitro and in vivo studies revealed that the knock down of Vps 35 exacerbates Aβ formation [99,100]. BDNF is an important neurotrophin that has been extensively studied to play a role in the pathogenesis of AD [101]. It has also been shown that the mutant huntingtin alters the retrograde transport of BDNF-tropomysoin related kinase B (TrkB) complexes in the striatal dendrites which subsequently cause neurodegeneration [102,103,104].

Altogether, these studies highlight the role of a dysregulated vesicular trafficking in the pathogenesis of prion and prion like disorders. In general, the endocytic pathway is affected, resulting in an impeded transport of the vesicular cargo to the lysosomes for degradation and to the retromer pathway, where the cargo is retrieved from early endosomes for subsequent transport to the Golgi complex or to the plasma membrane. Among the vesicular cargo are factors, such as BDNF, that are critical for neuronal function, suggesting similarities in the hypothesized mechanisms of neurodegeneration. Hence, such similarities offer the opportunities for exploring common potential therapeutic targets to develop medications for the treatment of these neurodegenerative disorders. A limitation of the above discussed studies is that most of them have been carried out in cell lines or utilized brains from terminal disease stages. Therefore, these results do not account for a potential progressive pattern of a dysfunction in the vesicular trafficking machinery, which appeals for a time-course study to correlate the onset of vesicular trafficking defects with neurodegeneration. An advantage of the cell culture models is that they allow to explore the direct consequences of protein aggregation on the physiology of neurons which is critical to define strategies to counteract such impairments. 

## 7. Therapeutic Strategies in Prion and Prion-Like Disorders

The pathogenicity and the infectivity of prion diseases are associated with the accumulation of PrP^Sc^ aggregates. One of the most promising strategies adopted are the methods that can prevent the interaction between the endogenous protein and its misfolded isoform. These include the down-regulation, re-routing, or intracellular retention of the cellular PrP^c^ itself via RNA interference or peptide aptamers [105,106,107]. Another approach is re-routing the PrP^c^ from the potential sites of conversion by using drugs, such as suramin, which retain them in the Golgi and activate post-ER quality control mechanisms [56]. Sulfated glycans, such as pentosan polysulfate (PPS) and dextran sulfate, can prevent the formation of PrP^Sc^ in cell culture models [108] and scrapie-infected mice [109]. On contrary, in cell-free conversion models, sulfated glycans, such as PPS, can stimulate the formation of PrP^Sc^ [110]. These conflicting results can be explained by the different effects PPS has on the PrP^c^ on the cell surface, as compared to the PrP^c^ in in vitro conversion. Among the effects that PPS has in cultured cells is that it enhances the endocytosis of PrP^c^, thereby reducing the amount of the surface localized PrP^c^ prone to conversion [111]. It also competitively inhibits the binding of PrP^c^ with other GAGs or cofactors that can aid in the pathogenic conversion [108]. However, in in vitro conversion assays, sulfated glycans appear to act as cofactors, favoring conversion by inducing a conformational change in PrP^c^ upon direct interaction [108]. Since lipid rafts are the main sites of PrP^c^ localization, using cholesterol lowering drugs, such as lovastatin [55] and squalestatin [112], or cholesterol binding drugs, like filipin [47] and amphotericin B [62], can abrogate proper raft formation and hence reduce PrP^Sc^ formation. 

Understanding the cellular biology of prion infection is crucial for devising therapeutic strategies for prion diseases. It has been demonstrated that prion propagation can only be sustained when the rate of PrP^Sc^ degradation does not exceed the rate of new conversion [113]. Autophagy is a major clearance mechanism of aggregated proteins in AD, PD, HD, and prion diseases [114], suggesting that stimulation of this pathway results in an enhanced degradation of the aggregated proteins. Treatment with resveratrol has shown to attenuate the aggregation of Aβ, α-synuclein, and mutant huntingtin in the animal models of AD, PD, and HD, respectively [115,116,117]. In prion diseases, a significant reduction in PrP^Sc^ aggregates has been attained by mammalian Target of rapamycin (mTOR) dependent or independent induction of autophagy by using small molecules, like imatinib mesylate [118,119], lithium, rapamycin, trehalose, and sirtuin [120,121,122]. Stimulating autophagy not only enhances PrP^Sc^ degradation but also prevents the exosomal release of PrP^Sc^ and hence the lateral transmission of prions to the neighboring cells [123].

An impairment seen in both prion and prion-like disorders is the trafficking defects in response to protein aggregation which possibly contribute to neurodegeneration. The retromer subunit Vps 35 is one of the most critical proteins involved in the retrograde trafficking. Vps 35 mutations or deficiencies have been implicated in several neurodegenerative diseases, such as AD, PD, and prion diseases. In mouse models of PD, an increased expression of Vps 35 reduces α-synuclein accumulation and also provide neuroprotection [124]. Pharmacological chaperones that stabilize the retromer complex and increase its half-life have been deployed as a therapeutic strategy to limit the pathogenic processing of amyloid precursor protein (APP) by directing it away from the endosomes in cultured hippocampal neurons [125]. Deficient levels of Vps 35 are also observed in prion diseases [86]. Reduced Vps 35 levels might serve as an impediment in the cell to degrade the PrP^Sc^ due to the vital role it plays in the lysosomal degradation pathway, as it is involved in the transport of lysosomal hydrolases, such as sortilin and cation independent 6 mannose phosphate receptor (CI-MPR) [126,127,128]. Vps 35 plays a major role in the trafficking of WASP and Scar homologue (WASH )complex and ATG9a protein, which has a role in the degradation pathways, such as macroautophagy [129]. It also regulates the chaperone mediated autophagy by retrieving the LAMP2A receptor to the lysosomes [130]. Hence, this calls for an active research in this unexplored area of the therapeutic potential of Vps 35 in prion diseases.

Ineffective lysosomal degradation is another important yet underexplored area regarding therapeutic solutions for these neurodegenerative diseases. Attenuated lysosomal degradation can be attributed to various factors, such as lysosomal rupture, due to the accumulation of α-synuclein aggregates in PD [131] or due to the incomplete maturation of the lysosomes [132]. Lysosomal maturation is associated with a decreasing pH gradient from early endosomes to lysosomes and also by their positioning in the neurons where they are more localized to the soma as compared to the distal parts of the axons [133]. Decreased lysosomal acidification related to Rab7 dysfunction has been reported in prion infection [85] and in early onset AD [134,135]. Abnormal transport and positioning of lysosomes have been implicated as one of the histopathological changes in AD and HD which might be a result of inefficient lysosomal maturation [91,136]. Since loss of LRRK2 or Rab7 also reduce Vps 35 levels in PD [96], it is reasonable to hypothesize that the loss of Rab7 could act as the upstream factor for the reduced levels of Vps 35 observed in AD and prion diseases. Along this line, it might be interesting to explore Rab7-based therapy as a potential treatment of these neurodegenerative diseases to combat impaired vesicular trafficking and lysosomal acidification. It has already been shown that cholesterol transport inhibiting amphiphiles, such as U18666A, reduce aberrant APP processing by retaining the localization of presinilins (PS) in Rab7-positive late endosomes. This shows that altering the sub-cellular cholesterol levels or Rab7 based therapeutic approaches to increase the transport of PS to Rab7-positive late endosomes can be adopted to reduce pathogenic Aβ processing [137]. Rab7 and Rab9 based approaches have already been adapted to correct lipid trafficking defects in Niemann-Pick type C (NP-C) lipid storage disease in fibroblast cell lines. By the over-expression of these proteins, it was possible to restore the normal trafficking of accumulated sphingolipids from the lysosomes to the Golgi [138]. Charcot-Marie Tooth 2B is an autosomal dominant neuropathy due to the partial loss of function of the Rab7 protein and stimulating enhanced Rab7 function may have utility for its treatment [139]. Rab7 based gene therapy approaches or small molecular interventions that can restore the normal physiological functions of the protein can be explored in the future as a cure to these above discussed diseases, including prion diseases [140].

## 8. Concluding Remarks

A very interesting point to ponder is whether the trafficking defects are the cause or an effect in these above discussed neurodegenerative diseases. In many spontaneous cases of prion diseases or AD, it appears that the amyloidogenic aggregates cause defects in trafficking without any obvious links to any known genetic mutations. On the other hand, many cases exist with a genetic link to the diseases, such as the mutation associated with LRRK2 in the late onset of PD; hence, it is a very pellucid fact that this defective mutation could affect vesicle trafficking and in fact trigger the onset of the disease, which subsequently results in the deposition of α-synuclein aggregates.

Regardless, manipulating, altering, or restoring the defective trafficking pathways in these neurodegenerative diseases is an arena that needs more active research, which could potentially open new doors to therapeutics and to understand the causal links of neurodegeneration associated with the vesicular trafficking defects.
